# Terlipressin versus placebo or noradrenalin in the treatment of hepatorenal syndrome: a systematic review and meta-analysis

**DOI:** 10.3389/fphar.2024.1418826

**Published:** 2024-09-04

**Authors:** Yue-Meng Wan, Song-Quan Huang, Hua-Mei Wu, Yu-Hua Li, Hong-Jing Yin, Ying Xu

**Affiliations:** ^1^ Gastroenterology Department II, The 2nd Affiliated Hospital of Kunming Medical University, Kunming, Yunnan Province, China; ^2^ Department of Hepatobiliary and Pancreatic Surgery, The Second Affiliated Hospital of Kunming Medical University, Kunming, Yunnan, China

**Keywords:** hepatorenal syndrome, vasoactive drugs, terlipressin, noradrenaline, treatment response, survival

## Abstract

**Background:**

Hepatorenal syndrome (HRS) bears a very poor prognosis with unmet need for safe and effective therapies. This systematic review and meta-analysis aimed to re-assess safety and efficacy of terlipressin versus placebo or noradrenaline for HRS, based on previous randomized controlled trials (RCTs).

**Methods:**

PubMed, EMBASE, MEDLINE (OvidSP) and Cochrane registers were searched for trials reporting HRS treatment by terlipressin or noradrenaline. Search terms included: “hepatorenal syndrome”, “terlipressin”, “noradrenaline”, and corresponding synonyms. Comparisons between terlipressin, noradreanaline, placebo and albumin were included. Meta-analysis was conducted for treatment response (both HRS reversal and complete response), mortality and adverse events.

**Results:**

15 RCTs were included, enrolling 1236 HRS patients (type 1: 1166, type 2: 70). Treatment with terlipressin+albumin resulted in significantly higher treatment response than placebo+albumin or albumin alone (risk ratio [RR]:2.75, 95% confidence interval [CI]:1.96 to 3.84; I^2^ = 28%, *p* = 0.23; n = 6). Noradrenaline was equally effective in treatment response compared to terlipressin (RR:1.19, 95% CI:0.96 to 1.46; I^2^ = 16%, *p* = 0.31; n = 7), but trials were limited by its non-blind design and small size. Sensitivity analysis showed no survival benefit with terlipressin compared to either placebo (RR:1.03, 95% CI:0.83 to 1.28; I^2^ = 0%, *p* = 0.72; n = 3) or noradreanline (RR:0.83, 95% CI:0.69 to 1.00; I^2^ = 4%, *p* = 0.39; n = 7) at 30 days of follow-up. Terlipressin carried higher risk of treatment-related adverse events compared to either placebo (RR:2.92, 95% CI:1.48 to 5.77; I^2^ = 0%, *p* = 0.75; n = 3) or noradrenaline (RR:2.45, 95% CI:1.37 to 4.37; I^2^ = 0%, *p* = 0.92; n = 5).

**Conclusion:**

Terlipressin is superior to placebo, and comparable to noradreanline in treatment response, but survival benefit is lacking. Noradrenaline, with low certainty, may be a better alternative for HRS.

## Highlights

• Terlipressin had significantly higher treatment response than placebo or albumin alone.
• Noradrenaline was equally effective in treatment response compared to terlipressin, but trials were limited by its non-blind design and small size.
• There was no survival benefit with terlipressin compared to either placebo or noradreanline at any follow-up time.

## 1 Introduction

Hepatorenal syndrome (HRS) is a severe form of acute kidney injury (AKI) with very poor prognosis (Moreau et al., 2013). It occurs frequently in decompensated cirrhosis, severe alcoholic hepatitis and fulminant liver failure (Gifford et al., 2017).The central mechanism underlying HRS is splanchnic arterial vasodilation that causes a decrease in the effective blood volume, and subsequent activation of vasoactive systems, including renin-angiotensin -aldosterone system, antidiuretic hormone and sympathetic nervous system, and ultimately severe renal vasoconstriction (Arroyo and Fernández, 2011; Angeli et al., 2015).

There are two distinct types of HRS, type 1 (HRS1) and type 2 HRS (HRS2). HRS1 is the most severe type of AKI, characterized by rapidly progressive kidney failure with a 2-week mortality rate reaching 80% (Martín-Llahí et al., 2011; Arroyo et al., 1996). HRS2 is a more insidious kidney failure, typically occurring in patients with refractory ascites, bearing a median survival of about 6 months without liver transplantation [Arroyo et al., 1996]. Recommended pharmacotherapy for HRS consists of discontinuation of diuretics and nephrotoxins, volume expansion with albumin, and vasoconstrictive drugs (Arora et al., 2020; Francoz et al., 2019).

Many randomized controlled trials (RCTs) have investigated the use of terlipressin or noradreanaline in combination with albumin for HRS treatment, which are also recommended in various guidelines (Biggins et al., 2021; Angeli et al., 2018; Bajaj et al., 2022) Due to scarcity of large head-to-head trials comparing terlipressin to norepinephrine for HRS treatment, previous systematic reviews have only found uncertain evidence with regard to their efficacy, safety, and survival benefit (Gifford et al., 2017; Best et al., 2019; Wang et al., 2018; Israelsen et al., 2017; Allegretti et al., 2017; Zheng et al., 2017; Mattos et al., 2016; Nassar Junior et al., 2014). Recently, some new RCTs have been published, especially the CONFIRM trial (Wong et al., 2021) and Singh et al. (Singh et al., 2023) that are not included in most reviews (Gifford et al., 2017; Best et al., 2019; Wang et al., 2018; Israelsen et al., 2017; Allegretti et al., 2017; Zheng et al., 2017; Mattos et al., 2016; Nassar Junior et al., 2014). Inclusion of these new trials may provide more precise estimates and improve the quality of evidence. Therefore, we conducted the present systematic review and meta-analysis to re-evaluate the safety and efficacy of terlipressin and noradrenaline for HRS management.

## 2 Materials and methods

We registered this systematic review and meta-analysis in the PROSPERO international prospective register of systematic reviews (CRD42024517812) and reported it according to the Preferred Reporting Items for Systematic Reviews and Meta-Analyses (PRISMA)(Moher et al., 2015).

### 2.1 Types of studies and patients

We considered eligible trials that reported on cirrhotic patients with HRS1, HRS2 or AKI-HRS treated by terlipressin or noradrenalin, published in English or Chinese language. Inclusion criteria were: 1) reporting adult patients aged >18 years with cirrhosis; 2) reporting patients’ baseline characteristics; 3) reporting treatment outcomes by terlipressin or noradrenalin; 4) reporting length of follow-up. Exclusion criteria were as follows: 1) non-RCT; 2) unclear diagnostic criteria of cirrhosis, AKI or HRS; 3) not reporting treatment response or mortality as endpoint; 4) presenting only survival analyses of events without specific number of patients; 5) reporting fewer than five patients; 6) inclusion of liver transplanted patients; 7) including patients from previous studies.

### 2.2 Study retrieval

Studies were retrieved by searching MEDLINE, EMBASE, Cochrane library and Pubmed databases. The following keywords were used (on 26 February 2024), with appropriate modification of the PubMed search strategy for other databases:

(((Hepatorenal syndrome [MeSH Terms]) OR ((Hepatorenal syndrome [Title/Abstract]) OR (Syndrome, Hepatorenal [Title/Abstract])))) AND ((((((telipressin [Title/Abstract]) OR (Terlypressin [Title/Abstract])) OR (Vasopressin [Title/Abstract])) OR (terlipressin [MeSH Terms]))) OR (((((Norepinephrine [Title/Abstract]) OR (noradrenalin [Title/Abstract])) OR (Levonorepinephrine [Title/Abstract])) OR (“Norepinephrine” [Mesh])))).

All identified studies were assessed independently for eligibility and inclusion by two reviewers (HM Wu and YH Li). Disagreements were resolved by discussion and consensus.

### 2.3 Data extraction and quality assessment

Two reviewers (Y Xu and HJ Yin) independently extracted relevant data from included trials: patient’s age and gender, aetiology of cirrhosis, Child-Pugh score and class, model for end-stage liver disease (MELD) score, baseline laboratory findings, complications of cirrhosis, type of HRS, death, reversal or progression of HRS, and adverse events. Two authors (YM Wan and SQ Huang) independently evaluated the risk of bias. The risk of bias of each trial was reported as “low risk”, “unclear risk”, or “high risk” using the Cochrane risk of bias assessment tool. Discordant results were resolved by consensus or arbitration (SQ Huang).

### 2.4 Outcome measures

The cumulative proportions of patients who experienced treatment response (including HRS reversal and complete response, since both were defined the same by various RCTs), partial response, all-cause mortality, treatment-related adverse events (AEs), serious adverse events (SAEs), and ischaemic adverse events (IAEs) were documented during follow up. To obtain more homogeneous estimates, the end of treatment (EOT), 30-, 60- and 90-day all-cause mortality rates were recorded accordingly. Data of outcomes of interest were all based on intention-to-treat analysis that might deviate from some previous reviews.

### 2.5 Data analysis

The analysis was performed with Review Manager (version 5.3; Cochrane Inc.). The expected heterogeneity between studies was quantified using the I^2^ statistic with I^2^ <25% indicating low heterogeneity, 25%–50% moderate and >50% I^2^ high heterogeneity. We performed pairwise meta-analysis using a random (I^2^ ≥ 50%) or fixed effects (I^2^<50%) model to calculate pooled estimates of risk ratios (RRs) and 95% confidence intervals (CIs) for the evaluation of treatment response and mortality outcomes (intention-to-treat data). Additionally, we conducted sensitivity or subgroup analysis to evaluate the potential source of heterogeneity by including only trials of low risk of bias.

## 3 Results

### 3.1 Study and patient characteristics

A total of 953 papers were identified through literature search. 831 papers were removed due to duplicates or ineligibility after screening titles and abstracts (case report, review, meta-analysis, editorial comments), leaving 122 full-text articles. A further 107 articles were eliminated after reviewing the contents due to the following reasons: single-armed cohort study (n = 51); terlipressin compared to other vasoconstrictors (n = 19); HRS compared to other type of renal failure (n = 13); re-analysis of previously published trials (n = 10); endpoint is not treatment response (n = 6); absence of baseline data for included patients (n = 5) and not RCT in study design (n = 3).

**FIGURE 1:**
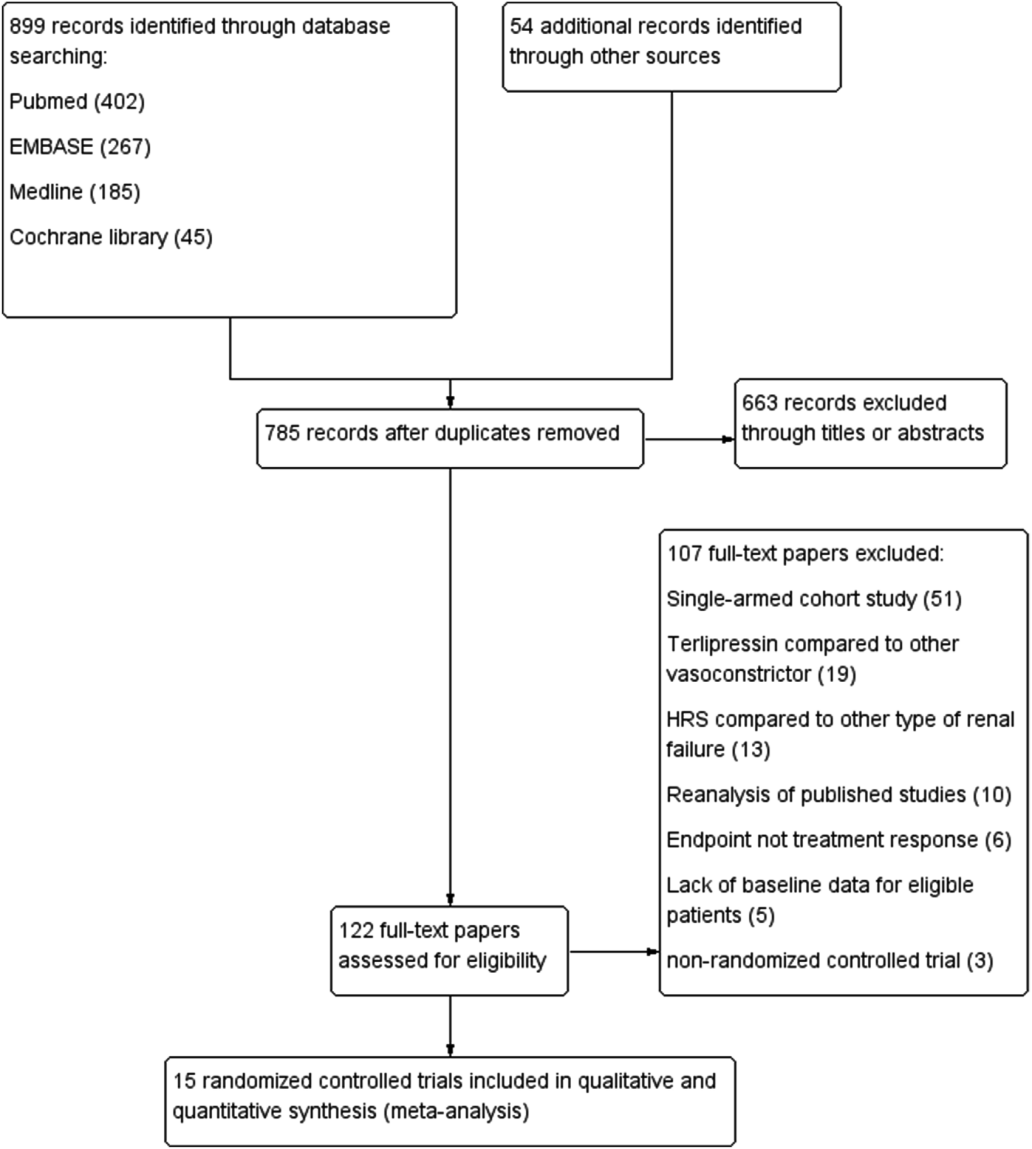
Flow chat of study selection.

Finally, a total of 15 RCTs were included in the present review (Figure 1) (Wong et al., 2021; Singh et al., 2023; Arora et al., 2020; Boyer et al., 2016; Cavallin et al., 2016; Saif et al., 2018; Singh et al., 2012; Goyal et al., 2016; Sharma et al., 2008; Sanyal et al., 2008; Neri et al., 2008; Solanki et al., 2003; Alessandria et al., 2007; Martín-Llahí et al., 2008; Ghosh et al., 2013). In all trials, albumin was administered to patients in both the terlipressin and comparator groups. One trial used both albumin and fresh frozen plasma in both study groups (Solanki et al., 2003). Two trials recruited both HRS1 and HRS2 patients (Alessandria et al., 2007; Martín-Llahí et al., 2008) and one enrolled only HRS2 patients [Ghosh et al., 2013]. HRS1 and HRS2 patients were randomized independently in these trials.

The study and patient characteristics are shown in Tables 1, 2, respectively. A total of 1236 patients with HRS (HRS1: 1166, HRS2: 70) were included in our meta-analysis. The dose of terlipressin varied between 0.5 and 2 mg/6 h and 1–2 mg/4 h, with a maximum dose 8–12 mg/day in all trials, and terlipressin was administered by intravenous bolus (IVB) in 11 studies and by continuous intravenous infusion (CIV) in four studies. In trials investigating the efficacy of noradrenaline, the dose was 0.5–3 mg/h or 0.1–0.7 μg/kg/min, and it was administered by CIV in all trials. Treatment duration was within 14 or 16 days. The albumin dose were between 20 and 60 g/day. Furthermore, the follow-up period varied between 15 and 180 days.

**TABLE 1:** Study characteristics.

Studies	Center/country	Terlipressin regimen	Comparator regimen	Albumin Regimen^&^	Treatment duration (d)	Followup period
Arora et al. (2020)	Single (India)	CIV 2–12 mg/24 h	NAD (0.5–3 mg/h, CIV)	60 g/d	≤14	28d
Wong et al. (2021)	Multicentric (USA/Canada)	IVB, 1–2 mg/5.5–6.5 h	Placebo	1 g/kg to 100 g × d1 + 20–40 g/d	≤14	90d
Boyer et al. (2016)	Multicentric (USA/Canada)	IVB, 1–2 mg/6 h	Placebo	20–40 g/d	≤16	90d
Cavallin et al. (2016)	Single (Italy)	CIV, 2–12 mg/24 h	Terlipressin (0.5–2 mg/4 h IVB)	1 g/kg to 100 g × d1 + 20–40 g/d	≤15	90d
Singh et al. (2023)	Single (India)	CIV, 2–12 mg/24 h	Terlipressin (2 mg/24 h) + NAD (0.5–3 mg/h), CIV	20 g/d	≤15	30d
Saif et al. (2018)	Single (India)	IVB, 0.5–2 mg/6 h	NAD (0.5–3 mg/h, CIV)	20–40 g/d	≤14	90d (stated 30d)
Singh et al. (2012)	Single (India)	IVB, 0.5–2 mg/6 h	NAD (0.5–3 mg/h, CIV)	20 g/d	≤15	30d
Goyal et al. (2016)	Single (India)	IVB, 0.5–2 mg/6 h	NAD (0.5–3 mg/h, CIV)	20 g/d	≤14	14d
Sharma et al. (2008)	Single (India)	IVB, 0.5–2 mg/6 h	NAD (0.5–3 mg/h, CIV)	20–40 g/d	≤15	30d
Sanyal et al. (2008)	Multicentric (USA/Germany/Russia)	IVB 1–2 mg/6 h	Placebo	100g × d1+ 25 g/d	≤14	180d
Neri et al. (2008)	Single (Italy)	IVB 1 mg/8 h × 5 d + 0.5 mg/8 h × 14d	Alb	100g × d1+ 20–40 g/d × 13d	≤19	90d
Solanki et al. (2003)	Single (India)	CIV, 1 mg/12 h	Placebo (1 mL/12 h, CIV)	20 g/d+FFP	≤15	15d
Alessandria et al. (2007)	Single (Italy)	IVB, 1–2 mg/4 h	NAD (0.1–0.7 μg/kg/min, CIV)	35–75 g/d	≤15	180d
Martín-Llahí et al. (2008)	Multicentric (Spain)	IVB, 1–2 mg/4 h	Alb	1 g/kg × d1 + 40 g/d	≤15	90d
Ghosh et al. (2013)	Single (India)	IVB, 0.5–2 mg/6 h	NAD (0.5–3 mg/h, CIV)	20 g/d	≤15	90d

Note: Alb, albumin; CIV, continuous intravenous infusion; FFP, fresh frozen plasma; HRS, hepatorenal syndrome; IVB, intravenous bolus; NAD, noradrenalin; NR, not reported.

^&^Albumin was given through intravenous infusion in both groups, if not stated otherwise.

**TABLE 2:** Characteristics of 1236 patients with hepatorenal syndrome included in this meta-analysis.

Trials	Age (yr)		Alcohol (%)		CTP score		MELD score		sCr (mg/dL)	
	Terli	Ctl	Terli	Ctl	Terli	Ctl	Terli	Ctl	Terli	Ctl
Wong et al. (2021)	54.0 ± 11.3	53.6 ± 11.8	67	66	10.0 ± 1.9	10.2 ± 1.9	32.7 ± 6.6	33.1 ± 6.2	3.5 ± 1.0	3.5 ± 1.1
Singh et al. (2023)	51.4 ± 9.9	49.6± 8.3	60	63.3	10.3 ± 1.6	10.7 ± 1.5	NR	NR	4.0 ± 1.3	4.2 ± 1.3
Arora et al. (2020)	40.3 ± 6.3	38.8 ± 7.0	73	71.7	11.0 ± 1.1	11.1 ± 1.1	33.3 ± 5.0	33.8 ± 5.0	2.7 ± 1.2	3.0 ± 1.1
Boyer et al. (2016)	55.8 ± 8.4	54.8 ± 8.5	50.5	54.5	10.4 ± 1.8	10.3 ± 1.7	33.5 ± 6.2	32.6 ± 5.5	3.6 ± 1.1	3.7 ± 1.1
Cavallin et al. (2016)	57.4 ± 10.5	59.4 ± 8.9	NR	NR	10.8 ± 2.1	10.8 ± 1.7	29.3 ± 7.8	29.8 ± 6.4	3.4 ± 1.3	3.1 ± 1.0
Saif et al. (2018)	53.8 ± 8.6	51.5 ± 12.8	NR	NR	11.9 ± 1.4	12.0 ± 1.3	29.1 ± 5.8	30.4 ± 9.2	3.2 ± 0.9	3.3 ± 1.1
Singh et al. (2012)	51.4 ± 11.6	48.3 ± 11.6	43.3	52.1	10.7 ± 2.0	10.4 ± 1.7	26.4 ± 3.1	24.7 ± 5.3	3.3 ± 0.7	3.1 ± 0.7
Goyal et al. (2016)	56.9 ± 6.1	54.7 ± 6.6	75	61.9	10.9 ± 1.7	10.8 ± 1.8	30.1 ± 5.9	29.2 ± 6.1	3.4 ± 1.6	3.1 ± 1.5
Sharma et al. (2008)	47.8 ± 9.8	48.2 ± 13.4	70	60	10.6 ± 0.8	11.0 ± 0.9	29.6 ± 6.2	31.6 ± 6.0	3.0 ± 0.5	3.3 ± 1.3
Sanyal et al. (2008)	50.6 ± 10.5	52.9 ± 11.4	51.8	51.8	11.7 ± 1.9	11.2 ± 1.8	33.4 ± 6.0	33.4 ± 6.3	3.96 ± 2.2	3.85 ± 1.2
Neri et al. (2008)	59 ± 4	60 ± 3	11.2	15.4	11.5 ± 1	11.2 ± 0.8	NR	NR	2.8±1.1	2.9±1.2
Solank et al. (2003)	51.0 ± 5.0	52.0 ± 4.8	NR	NR	NR	NR	NR	NR	2.9 ± 0.1	2.2 ± 0.2
Alessandria et al. (2007)	55 ± 2	56 ± 3	33.3	20	11 ± 1	10 ± 1	26 ± 2	26 ± 1	2.5 ± 0.3	2.3 ± 0.2
Martín-Llahí et al. (2008)	59 ± 10	55 ± 11	61	83	10 ± 2	11 ± 2	30 ± 9	28 ± 8	3.6 ± 1.4	4.1 ± 2.4
Ghosh et al. (2013)	45.8 ± 9.2	48.2 ± 10.5	65.2	69.5	10.0 ± 1.8	10.5 ± 2.4	21.3 ± 2.8	21.0 ± 3.3	2.2 ± 0.2	2.1 ± 0.2

Note: CTP, Child-Turcotte-Pugh; MELD, model for end-stage liver disease; NR, not reported; sCr, serum creatinine; Terli, terlipressin group; Ctl, comparator group.

### 3.2 Risk of selection bias

**FIGURE 2:**
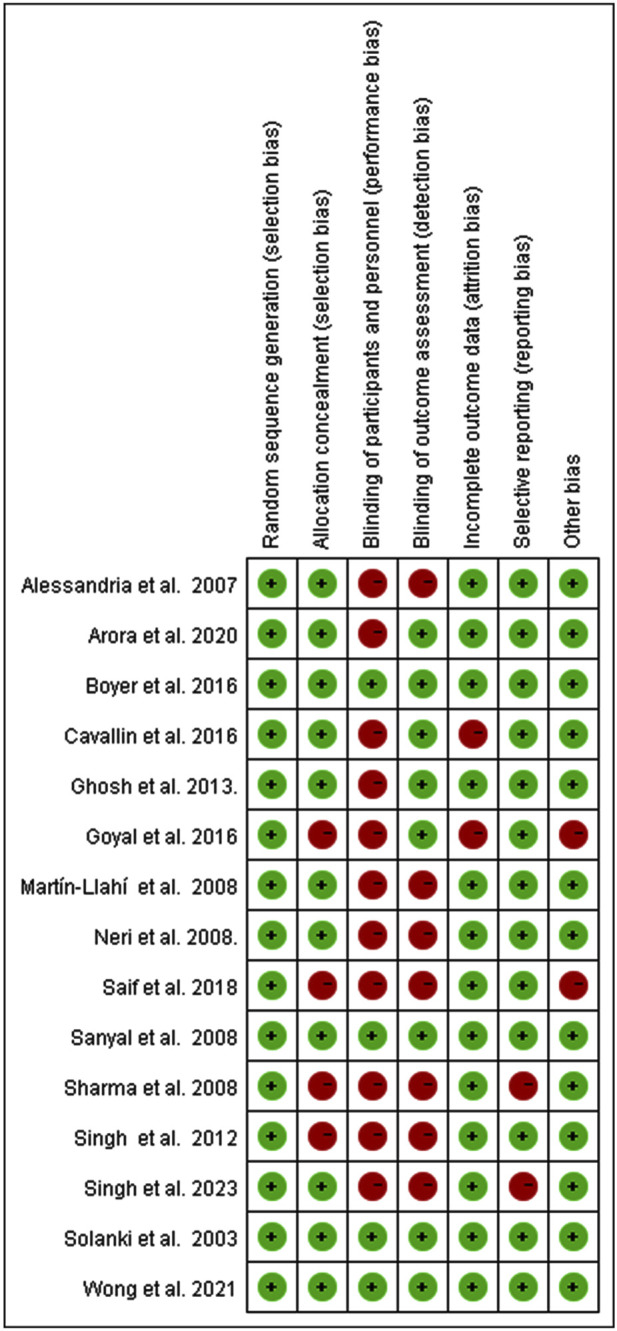
Risk of bias summary.

Evaluation of Cochrane risk of bias is presented in Figure 2. In our series, three trials were double-blinded (20%) (Wong et al., 2021; Boyer et al., 2016; Sanyal et al., 2008), one was single-blinded (6.7%) (Solanki et al., 2003), and the remaining 11 trials were not blinded (73.3%). Among the included trials, 26.7% were judged to have high risk of bias for allocation concealment, 73.3% for non-blinding of participants and personnel, 46.7% for non-blinding of outcome assessment, 16.6% for incomplete outcome data, and selective reporting or other bias. 53.3% of trials reported a sample size calculation.

### 3.3 Results of data analysis by comparison

The definitions of HRS reversal were similar across studies (Supplementary Table S1). Seven trials defined HRS reversal as a decrease in serum creatinine (sCr) to <1.5 mg/dL with treatment, and eight trials did not report it. Nine trials defined complete response as a decrease in sCr to <1.5 mg/dL, two trials defined it as the return of sCr to a value within 0.3 mg/dl of the baseline (Arora et al., 2020; Wong et al., 2021), and four only reported HRS reversal as a decrease in sCr to <1.5 mg/dL instead (Boyer et al., 2016; Saif et al., 2018; Sanyal et al., 2008; Solanki et al., 2003). Two trials reported HRS reversal without defining it (Arora et al., 2020; Solanki et al., 2003). In our series, HRS reversal and complete response were combined as one indicator, since sCr value ≤ 1.5 mg/dL was commonly used to define both across trials.

As for AEs, one trial did not report the information at all (Saif et al., 2018), one trial presented the sum of AEs for all patients combined (Neri et al., 2008), and another one simply stated that most patients treated with terlipressin had transient abdominal cramps and water diarrhea (Alessandria et al., 2007). None of the included trials reported definition of SAEs, so we considered treatment-related SAEs as those causing discontinuation of treatment. One trial stated that IAEs included abdominal pain, intestinal ischemia, cyanosis, vascular skin disorders, and pulmonar edema (Boyer et al., 2016). So we considered abdominal pain, chest pain, myocardial infarction and finger or toe cyanosis together as IAEs.

### 3.4 Terlipressin versus placebo or albumin alone

**FIGURE 3:**
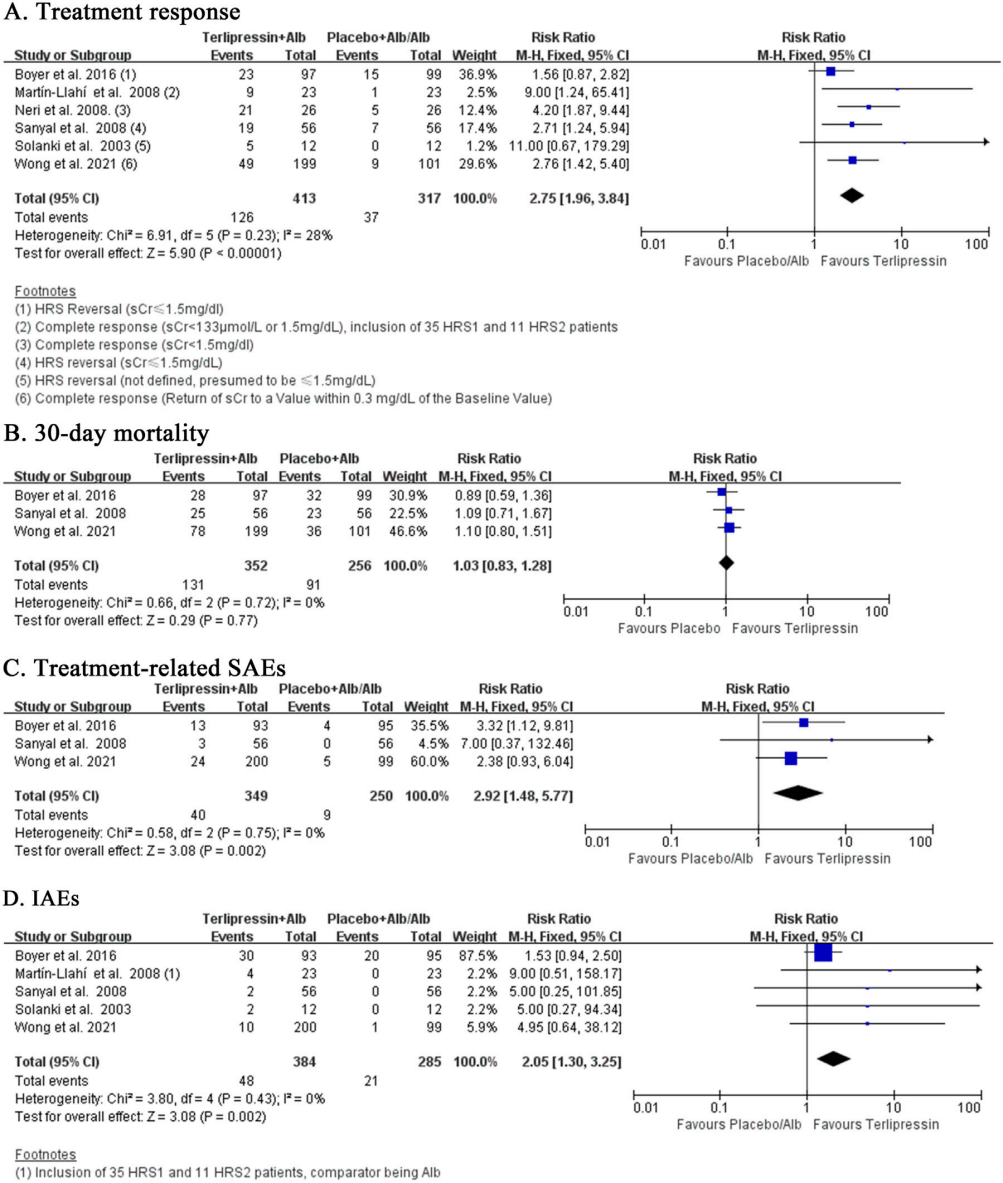
**(A–D)** Terlipressin versus Placebo or Albumin alone.

Compared to placebo or albumin alone, terlipressin had significantly higher treatment response (RR:2.75, 95% CI:1.96 to 3.84; I^2^ = 28%, *p* = 0.23; n = 6; Figure 3A). Sensitivity analysis confirmed this by including only trials of low risk of bias and low heterogeneity (RR:2.35, 95% CI:1.61 to 3.44; I^2^ = 11%, *p* = 0.34; n = 4; Supplementary Figure S1A). Subgroup analysis was the same when comparator was restricted to placebo (RR:2.35, 95% CI 1.61 to 3.44; I^2^ = 11%, *p* = 0.34; n = 4; Supplementary Figure S1A), but was more robust when restricted to albumin alone (RR:5.00, 95% CI:2.34 to 10.70; I^2^ = 0%, *p* = 0.47; n = 2; Supplementary Figure S1B). Notably, Wong et al. (Wong et al., 2021) reported both complete response (defined as return of sCr to a value within 0.3 mg/dL of the baseline value) and HRS reversal (defined as a sCr ≤1.5 mg/dL) data. When using data of HRS reversal (72/199 vs. 17/101; RR:2.48, 95% CI: 1.84 to 3.34, I^2^ = 30%, *p* = 0.21; n = 6; Supplementary Figure S2A) in Wong et al. (Wong et al., 2021), repeated analysis was slightly weaker than using complete response (49/199 vs. 9/101; RR:2.75, 95% CI:1.96 to 3.84; I^2^ = 28%, *p* = 0.23; n = 6; Figure 3A). Repeated subgroup or sensitivity analysis was similar when comparator was restricted to only placebo but complete response data was changed to HRS reversal in Wong et al. (Wong et al., 2021) (RR: 2.35 vs. 2.14; Supplementary Figures S1A, S2B). Moreover, four trials reported partial response (defined as either regression of AKI stage plus sCr≥0.3 mg/dl above baseline or sCr >1.5 mg/dL plus decrease ≥50% baseline value) (Wong et al., 2021; Sanyal et al., 2008; Neri et al., 2008; Martín-Llahí et al., 2008). Analysis of overall response (complete and partial response) also favoured terlipressin over placebo or albumin alone (RR:1.93, 95% CI:1.49 to 2.51; I^2^ = 17%, *p* = 0.31; n = 4; Supplementary Figure S2C), although both treatments were not different in partial response (RR:0.89, 95% CI:0.55 to 1.42; I^2^ = 32%, *p* = 0.22; n = 4; Supplementary Figure S2D).

Despite superior efficacy, terlipressin failed to reduce the mortality risk at 30 days of follow-up compared to placebo (RR:1.03, 95% CI:0.83 to 1.28; I^2^ = 0%, *p* = 0.72; n = 3; Figure 3B) in three trials of low risk of selection bias and low heterogeneity. Repeated analysis obtained similar results at the EOT (RR:0.95, 95% CI:0.71 to 1.27, I^2^ = 34%, *p* = 0.21; n = 4; Supplementary Figure S3A), 60 days (RR:1.00, 95% CI:0.83 to 1.20; I^2^ = 0%, *p* = 0.46; n = 3; Supplementary Figure S3B) and 90 days (RR:1.02, 95% CI: 0.86 to 1.22; I^2^ = 0%, *p* = 0.51; n = 3; Supplementary Figure S3C). In addition, two more trials comparing terlipressin to albumin alone also reported the mortality rates at 90 days [Neri et al., 2008; Martín-Llahí et al., 2008]. Meta-analysis of these five trials still showed no survival benefit at 90 days for terlipressin (RR:0.97, 95% CI:0.83 to 1.12; I^2^ = 42%, *p* = 0.14; n = 5; Supplementary Figure S3D).

Terlipressin tended to increase the risk of treatment-related AEs (RR:1.46, 95% CI:0.95 to 2.26; I^2^ = 71%, *p* = 0.02; n = 4; Supplementary Figure S4A) compared to placebo or albumin alone, though the heterogeneity was very high. Sensitivity analysis including only trials with low risk of bias significantly boosted this relationship (RR:2.92, 95% CI:1.48 to 5.77; I^2^ = 0%, *p* = 0.75; n = 3; Figure 3C) for treatment-related SAEs. Further analysis of IAEs also maintained this significant relationship (RR:2.05, 95% CI:1.30 to 3.25; I^2^ = 0%, *p* = 0.43; n = 5; Figure 3D), which was slightly weakened in sensitivity analysis by including only trials with low risk of selection and performance bias (RR:1.90, 95% CI:1.19 to 3.02; I^2^ = 0%, *p* = 0.49; n = 4; Supplementary Figure S4B).

### 3.5 Terlipressin versus noradrenaline

**FIGURE 4:**
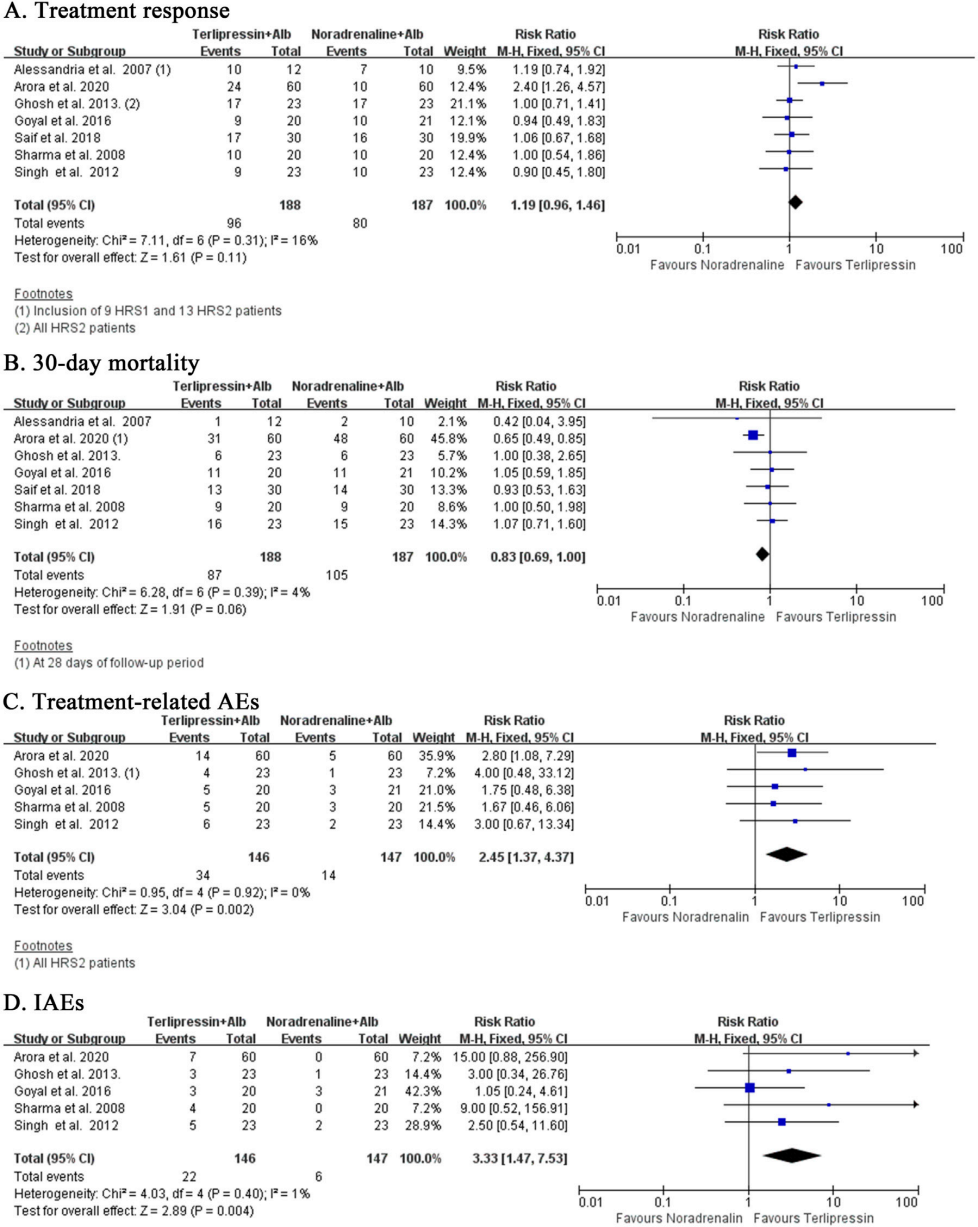
**(A–D)** terlipressin versus noradrenaline.

Terlipressin and noradrenaline showed equal efficacy with regard to treatment response (RR:1.19, 95% CI:0.96 to 1.46; I^2^ = 16%, *p* = 0.31; n = 7; Figure 4A). Subgroup analysis including only HRS1 patients (RR:1.23, 95% CI:0.95 to 1.59; I^2^ = 23%, *p* = 0.26; n = 6; Supplementary Figure S4C) or HRS2 patients (RR:1.06, 95% CI: 0.78 to 1.43; I^2^ = 0%, *p* = 0.50; n = 2; Supplementary Figure S4D) achieved similar findings. Mortality rate with terlipressin tended to be lower than noradrenaline at 30 days (RR:0.83, 95% CI:0.69 to 1.00; I^2^ = 4%, *p* = 0.39; n = 7; Figure 4B) with low heterogeneity. Subgroup analysis eliminating trials with HRS2 patients revealed almost the same results at the EOT but with high heterogeneity (RR:0.85, 95% CI: 0.54 to 1.36; I^2^ = 67%, *p* = 0.03; n = 4; Supplementary Figure S5A). Repeated analysis inclusive of HRS2 patients showed similar findings at the EOT (RR:0.84, 95% CI: 0.56 to 1.27; I^2^ = 56%, *p* = 0.06; n = 5; Supplementary Figure S5B), 60 days (RR:0.83, 95% CI: 0.58 to 1.19; I^2^ = 0%, *p* = 0.58; n = 2; Supplementary Figure S5C) and 90 days (RR:0.86, 95%CI:0.68 to 1.10; I^2^ = 0%, *p* = 0.84; n = 3; Supplementary Figure S5D). Interestingly, terlipressin had significantly higher risk of treatment-related AEs when compared to noradrenaline (RR:2.45, 95% CI:1.37 to 4.37; I^2^ = 0%, *p* = 0.92; n = 5; Figure 4C). This relationship was slightly weakened after eliminating HRS2 patients (RR:2.33, 95% CI:1.28 to 4.25; I^2^ = 0%, *p* = 0.56; n = 4). Terlipressin also induced more IAEs than noradrenaline (RR:3.33, 95% CI:1.47 to 7.53; I^2^ = 1%, *p* = 0.40; n = 4; Figure 4D).

### 3.6 Terlipressin versus noradrenaline combined with terlipressin

In our series, only one trial investigated the use of terlipressin combined with noradrenaline for HRS (Singh et al., 2023). The results showed that terlipressin combined with noradrenaline was beneficial with regard to complete response (RR:0.65, 95% CI:0.43 to 0.98, *p* = 0.04; Supplementary Figure S6A), but not with regard to mortality at either the EOT (RR:1.60, 95% CI:0.59 to 4.33, *p* = 0.36; Supplementary Figure S6B) or 30 days (RR:1.36, 95% CI:0.85 to 2.17, *p* = 0.20; Supplementary Figure S6C). Interestingly, terlipressin combined with noradrenaline tended to reduce treatment-related AEs (RR: 2.75, 95% CI:0.99 to 7.68, *p* = 0.05; Supplementary Figure S6D), compared to terlipressin alone.

### 3.7 Terlipressin CIV versus terlipressin IVB

Cavallin et al. (2016) investigated the use of terlipressin by CIV or IVB for HRS patients, and showed that HRS reversal (RR:1.22, 95% CI:0.77 to 1.93, *p* = 0.40; Supplementary Figure S7A) or 90-day mortality (RR:1.58, 95% CI:0.86 to 2.91, *p* = 0.14; Supplementary Figure S7B) were not significantly different between the two routes of therapy. Nonetheless, terlipressin CIV therapy tended to reduce risk of treatment-related AEs (RR:0.48, 95% CI:0.22 to 1.01, *p* = 0.05; Supplementary Figure S7C), rather than IAEs (RR:0.73, 95% CI:0.22 to 2.35, *p* = 0.59; Supplementary Figure S7D), compared to IVB therapy.

## 4 Discussion

Our review presents an updated evidence regarding the comparative efficacy and safety of terlipressin and noradrenaline for HRS. We confirmed with high certainty of evidence that terlipressin had significantly better treatment response, but lacked survival benefit compared to placebo or albumin alone. We also confirmed with low certainty of evidence that terlipressin was equally effective in treatment response, but tended to reduce 30-day mortality compared to noradrenaline. Moreover, terlipressin was associated with markedly higher risk of treatment-related SAEs (compared to placebo), AEs (compared to noradrenaline), and IAEs compared to either placebo or noradrenaline.

The CONFIRM trial published in 2021 is deemed the largest, multicentric, double-blinded RCT evaluating terlipressin compared to placebo up to date (Wong et al., 2021). As for treatment response, previous reviews before 2021 suggested superiority of terlipressin to placebo or albumin alone (Gifford et al., 2017; Wang et al., 2018; Zheng et al., 2017). Including data from the CONFIRM trial, our review further confirmed this with high certainty, because we provided solid sensitivity analysis by including only the three large, multicentric and double-blinded RCTs (Wong et al., 2021; Boyer et al., 2016; Sanyal et al., 2008), and repeated subgroup analyses by restricting comparator to either placebo or albumin alone. Since the CONFIRM trial reported data of both complete response and HRS reversal with different diagnostic criteria, we used both types of data to perform the meta-analysis that showed consistent results. All these analyses certified the superiority of terlipressin to placebo or albumin alone, which was in concord with a latest meta-analysis that also included the CONFIRM trial, but used different analytic method and included different trials (Pitre et al., 2022).

As for survival benefit, previous reviews are not consistent. Some reviews suggested no difference (Gifford et al., 2017; Zheng et al., 2017; Best et al., 2019), whereas others showed benefit of terlipressin over placebo or albumin alone. [13,15] Our meta-analysis demonstrated no survival benefit in terlipressin relative to placebo or albumin alone with high certainty, since our sensitivity analysis included the three high-quality RCTs with low risk of bias (Wong et al., 2021; Boyer et al., 2016; Sanyal et al., 2008). Moreover, we performed detailed subgroup analyses of mortality rates at EOT, 30 days, 60 days and 90 days, all of which suggested no difference between the two treatments. Interestingly, the latest meta-analysis found that terlipressin might reduce mortality compared with placebo with low certainty as the author acknowledged (Pitre et al., 2022). In contrast to this review by Pitre et al. (2022), we provided detailed subgroup and sensitivity analyses on the mortality rates at various time points using data from the high-quality trials, which may add credit to our review.

As for AEs, previous reviews were consistent with increased AEs in terlipressin compared to placebo or no intervention, although which type of AEs was not consistent. For example, Allegretti et al. (2017) showed that terlipressin increased the risk of cardiovascular events, but it had no effect on the risk of SAEs. Pitre et al. (2022) concluded that terlipressin might increase the risk of SAEs, while Gifford et al. (Gifford et al., 2017) reported that terlipressin was associated with increased risk of IAEs, but similar overall AEs. Our review found that terlipressin led to increased both treatment-related SAEs and IAEs compared to placebo or albumin alone. These inconsistent results may derive from varied definitions or reports for these events in clinical trials. Indeed, only one trial described the definition of IAEs (Boyer et al., 2016). Even in the CONFIRM trial, both acute respiratory failure and respiratory failure were reported confusingly (Wong et al., 2021).

Previous reviews found that terlipressin was equal to noradrenaline in efficacy and mortality, but inferior to noradreanline in incidence of SAEs and IAEs (Gifford et al., 2017, Best et al., 2019; Wang et al., 2018; Israelsen et al., 2017; Zheng et al., 2017; Mattos et al., 2016; Nassar Junior et al., 2014). With accrued new data from Arora et al. (Arora et al., 2020), our review reinforced previous ones, corroborating similar effects in efficacy and mortality between treatment with terlipressin and noradrenaline, but lower treatment-related SAEs and IAEs in terlipressin than in noradrenaline. Notably, albumin infusion can impact the effect of terlipressin for the treatment of HRS, thus compounding the side-effect profile seen with terlipressin (Ortega et al., 2002), which may be clarified by a well-conducted meta-analysis of the accrued doses of albumin used in the terlipressin arm versus placebo or noradrenaline arm. Nonetheless, it is difficult to perform such a meta-analysis, since few studies presented the accurate estimation of the accrued doses of used albumin.

Moreover, our review is also in keeping with another latest review by Olson and Subramanian, 2024 that also included Arora et al. (Arora et al., 2020) with regard to treatment response and mortality. However, our review is different from Olson and Subramanian, 2024 in the following aspects: first, the trial by Indrabi et al. (2013) was not included in our review due to absence of many important information, such as HRS diagnostic criteria, baseline serum creatinine and MELD score. Second, we included the trial by Ghosh et al. (2013) that enrolled only HRS2 patients, Singh et al. (2023) that investigated the combination of terlipressin and noradreanline for HRS, and Cavallin et al. (2016) that evaluated the use of terlipressin by CIV or IVB. Third, we performed detailed subgroup or sensitivity analyses comparing treatments in either HRS1 or HRS1 patients, and comparing the mortality rates at EOT, 30 days, 60 days and 90 days following terlipressin or noradrenaline treatment. Last, Olson and Subramanian, 2024 did not perform meta-analysis on the incidence of AEs. All these differences make our review necessary and invaluable.

In terms of medical costs, several studies indicated that treatment with noradrenaline costed less than that with terlipressin if only the costs of the vasoconstrictor drugs were evaluated (Singh et al., 2012; Sharma et al., 2008; Alessandria et al., 2007; Ghosh et al., 2013). Specifically, Alessandria et al. (Alessandria et al., 2007) reported that the average cost per patient was 107 ± 31 euros for noradrenaline and 1,536 ± 40 euros for terlipressin (*P*< 0.0001). For 15 days, Singh et al. (2012) reported a cost of 275 euros for noradrenaline and 975 euros for terlipressin (*P*< 0.05). Sharma et al. (Sharma et al., 2008) showed that the cost for noradrenaline at a dose of 1 mg/h/day was 750 US dollars as compared to 2,500 US dollars for terlipressin at a dose of 6 mg/day for 15 days (*P*< 0.05). Ghosh et al. (2013) also reported a significantly lower cost of 311 US dollars for noradrenaline at 11.3 mg/day than 804 US dollars for terlipressin at 1.9 mg/day for a 15-day course (*P*< 0.05). Nonetheless, considering only drug-related costs is not adequate, since terlipressin can be administered in regular wards (Nassar Junior et al., 2014; Martín-Llahí et al., 2008). In contrast, noradrenaline is usually given as a continuous infusion through a central venous catheter in the setting of intensive care unit that involves other associated costs (Angeli et al., 2018; Nassar Junior et al., 2014). Thus, Mattos et al. (2016) demonstrated that terlipressin was more cost-effective than noradrenaline when all direct medical costs involved in a hypothetical hospitalisation were calculated.

Interestingly, Singh et al. (2023) firstly reported the combination of terlipressin and noradrenaline for HRS treatment. This trial is limited by its single-centered, non-blinding design, small size and lack of allocation concealment (risk of bias). Nonetheless, this trial suggested fewer AEs but similar efficacy, which may provide some useful information for future trials. Up to date, only Cavallin et al. (2016) assessed the effects of different routes of terlipressin delivery to HRS patients in a RCT, and found similar efficacy, but lower daily effective dose and better tolerability by CIV. More high-quality randomized trials are needed to improve the certainty of evidence for findings in these two trials.

Our review has the following limitations. First, trials examining the efficacy of noradrenaline are relatively small, single-centered and non-blinded, and some trials did not report sample size calculations. Second, trials had some variation in diagnostic criteria for HRS, complete response or reversal, and unclear definition of SAEs or IAEs, which can impact the evaluation of outcomes. Indeed, Terres et al. (2022) suggested that the use of evidence-based protocols for the diagnosis and treatment of HRS could reduce cost and mortality in tertiary hospitals. Third, we included HRS2 patients. Nonetheless, our subgroup analysis showed no difference in treatment response between HRS1 and HRS2 patients. Moreover, the included trials showed no evidence of significant heterogeneity, and adopted similar treatment protocols, and there was no evidence suggesting differed response to vasoconstrictor treatment between HRS1 and HRS2 patients. Due to the scarcity of RCTs for HRS, we thus included the trials with HRS2 patients as previous reviews did (Gifford et al., 2017; Wang et al., 2018; Mattos et al., 2016; Olson and Subramanian, 2024). The strengths of our review are inclusion of the latest updated data, careful data collection (including HRS reversal, complete response and overall response), stringent meta-analysis (using both complete response and HRS reversal data from Wong et al. (2021), detailed subgroup analyses (including mortality rates at various time points, HRS1 and HRS2 patients and different comparators), and sensitivity analysis using only the three high-quality trials to examine the efficacy, mortality and safety of treatments, which can complement the shortness of previous reviews (Gifford et al., 2017, Best et al., 2019; Wang et al., 2018; Israelsen et al., 2017; Zheng et al., 2017; Mattos et al., 2016; Nassar Junior et al., 2014; Pitre et al., 2022; Olson and Subramanian, 2024).

## 5 Conclusion

Terlipressin is superior to placebo, and comparable to noradreanline in efficacy, but lacks survival benefit compared to either placebo or noradrenaline. Noradrenalin may be a better alternative for HRS patients due to similar efficacy but fewer treatment-related AEs and IAEs. More larger, better-designed trials are needed to improve the certainty of our findings.

## Data Availability

The original contributions presented in the study are included in the article/Supplementary Material, further inquiries can be directed to the corresponding author.

## Supplementary material

Supplementary Figure S1 (A, B) Sensitivity or subgroup analysis for treatment response comparing terlipressin to placebo or albumin alone.

Supplementary Figure S2 (A–D) Treatment response and subgroup analysis for comparing terlipressin to placebo or albumin alone.

Supplementary Figure S3 (A–D) Mortality rates for comparing terlipressin to placebo or albumin alone.

Supplementary Figure S4 (A–D) Adverse events for treatment with terlipressin, placebo, albumin alone, and treatment response for terlipressin versus noradrenaline.

Supplementary Figure S5 (A–D) Mortality rates for terlipressin versus noradrenaline.

Supplementary Figure S6 (A–D) Treatment for terlipressin versus noradrenaline plus terlipressin.

Supplementary Figure S7 (A–D) Terlipressin continuous intravenous infusion (CIV) versus terlipressin intravenous bolus (IVB).

## Abbreviations

AEs, adverse events; AKI, acute kidney injury; Alb, albumin; CIs, confidence intervals; CIV, continuous intravenous infusion; Ctl, comparator group; CTP, Child-Turcotte-Pugh; EOT, end of treatment; FFP, fresh frozen plasma; HRS, Hepatorenal syndrome; HRS1, type-1 HRS; HRS2, type-2 HRS; IAEs, ischaemic adverse events; IVB, intravenous bolus; MELD, model for end-stage liver disease; NAD, noradrenalin; NR, not reported; PRISMA, the Preferred Reporting Items for Systematic Reviews and Meta-Analyses; RCTs, randomized controlled trials; RR, risk ratios; SAEs, serious adverse events; sCr, serum creatinine; Terli, terlipressin group.
