# Supplementary material for: Terlipressin versus placebo or noradrenalin in the treatment of hepatorenal syndrome: a systematic review and meta-analysis
Source: Front Pharmacol. 2024 Sep 4;15:1418826. doi: 10.3389/fphar.2024.1418826 (PMC11408352; doi:10.3389/fphar.2024.1418826)
Supplement: Supplementary file 2 [file Table1.docx]

Supplementary Table 1 Definitions in eligible studies.

| Studies | HRS definition | HRS reversal | CR | PR |
| --- | --- | --- | --- | --- |
| Arora  (2020) | ICA-2015 criteria: HRS-AKI [1] | Not clarified | sCr≤0.3 mg/dl of the baseline | regression of AKI stage plus sCr≥0.3mg/dl above baseline |
| Wong  (2021) | **HRS1:**  Rapidly,progressive kidney failure, with a doubling of the  sCr≥2.25mg/dl (199μmol/l) within 14 days before randomization. | **Verified reversal:**  2 consecutive sCr ≤1.5 mg/dL  at least 2 hours apart, while on  treatment (up to 24 hours after the last dose) by Day 14 or discharge and excluding sCr values after transplant, RRT, TIPS, and open label vasopressors.  **Reversal:** sCr<1.5mg/dL with treatment. | sCr≤0.3 mg/dl of the baseline, | regression of AKI stage plus sCr≥0.3mg/dl above baseline. |
| Boyer  (2016) | ICA-2007 criteria[2]  sCr to ≥2.5 mg/dL within 2 weeks. | **CHRSR:**  2 sCr values ≤1.5mg/dL, collected at least 40h apart (48 hours minus an 8h window) while on treatment, defined as within 24 hours of the last dose of study treatment, and without RRT or liver transplant;  **Reversal:** at least 1 sCr≤1.5 mg/dL while on treatment. | Not defined | Not defined |
| Cavallin  (2016) | ICA-2007criteria[2]  sCr to ≥2.5 mg/dL within 2 weeks. | Not defined | ≤133 µmol/L  (≤1.5mg/dL) | sCr >1.5mg/dL, but decrease  ≥50% baseline value. |
| Singh  (2023) | ICA-2007 criteria[2] | Not defined | sCr< 1.5mg/dl | Not defined |
| Saif  (2018) | **HRS1:** cirrhosis with ascites; sCr> 133μmol/L(1.5mg/dL); no improvement of sCr (decrease to a level of ≤ 133 μmol/L) after at least 2 days of diuretic withdrawal and volume expansion with albumin; the dose of albumin used was 1g/kg of body weight per day up to a maximum of 100 g/d for at least 2 days; absence of shock; no current or recent treatment with nephrotoxic drugs; absence of parenchymal kidney disease (as indicated by proteinuria > 500 mg/d, microhematuria as evidenced by > 50 red blood cells per high-power field, and/or abnormal renal ultrasonography). | sCr≤1.5 mg/dL | Not defined | Not defined |
| Singh  (2012) | ICA-2007 criteria[2]  sCr to ≥2.5 mg/dL within 2 weeks. | Not defined | sCr<1.5 mg/dL | Not defined |
| Goyal  (2016) | ICA-2007 criteria[2]  sCr to ≥2.5 mg/dL within 2 weeks. | sCr≤1.5 mg/dL | sCr≤1.5 mg/dL | sCr >1.5mg/dL, but decrease  ≥50% baseline value. |
| Sharma  (2008) | ICA-2007 criteria[2]  sCr to ≥2.5 mg/dL within 2 weeks. | sCr≤1.5 mg/dL | sCr≤1.5 mg/dL | sCr >1.5mg/dL, but decrease  ≥50% baseline value. |
| Sanyal  (2008) | ICA-2007 criteria[2]  sCr to ≥2.5 mg/dL within 2 weeks. | **Treatment success:**  sCr≤1.5mg/dL on 2 occasions at least 48 hours apart, without dialysis, death, or recurrence of HRS type 1 on or prior to day 14.  **Reversal:**  SCr≤1.5mg/dL without dialysis. | Not reported | sCr decreased by>50% from baseline but not ≤1.5mg/dL, without dialysis or recurrence of HRS. |
| Neri  (2008) | ICA-2007 criteria[2]  sCr to ≥2.5 mg/dL within 2 weeks. | Not defined | sCr≤1.5mg/dL | sCr >1.5mg/dL, but decrease  ≥50% baseline value. |
| Solanki  (2003) | ICA-2007 criteria[2]  sCr to ≥2.5 mg/dL within 2 weeks. | Reported reversal (presumed to be sCr≤1.5mg/dL) | Not clarified | Not clarified |
| Alessandria  (2003) | ICA-1996 criteria[3] | sCr≤1.5mg/dL  during the treatment | sCr≤1.5mg/dL  during the treatment | sCr >1.5mg/dL, but decrease  ≥30% baseline value. |
| Martín  (2008) | ICA-1996 criteria[3] | Not defined | sCr≤1.5mg/dL  during the treatment | sCr >1.5mg/dL, but decrease  ≥50% baseline value. |
| Gosh  (2013) | ICA-2007 criteria[2]  1.5mg/dL< sCr to <2.5 mg/dL | Not defined | sCr<1.5mg/dL | Not defined |

Note: AKI, acute kidney injury; CHRSR, Confirmed HRS Reversal; CR, Complete Response; ICA, International Club of Ascites; HRS, hepatorenal syndrome; PR, Partial Response; RRT, Renal Replacement Therapy; sCr, serum creatine. [1], Gut. 2015; 64(4):531-7. [2], Gut. 2007;56(9):1310-8. [3], Hepatology. 1996; 23(1):164-76.
